# Fibered Confocal Fluorescence Microscopy for the Noninvasive Imaging of Langerhans Cells in Macaques

**DOI:** 10.1155/2017/3127908

**Published:** 2017-06-07

**Authors:** Biliana Todorova, Nina Salabert, Sabine Tricot, Raphaël Boisgard, Mélanie Rathaux, Roger Le Grand, Catherine Chapon

**Affiliations:** ^1^CEA, Université Paris Sud 11, INSERM U1184, Immunology of Viral Infections and Autoimmune Diseases Research Center, IDMIT Infrastructure, 92265 Fontenay-aux-Roses, France; ^2^Vaccine Research Institute (VRI), Créteil, France; ^3^CEA, Institute of Biomedical Imaging (I2BM), DSV/I2BM/SHFJ/INSERM U1023, CEA, Orsay, France

## Abstract

**Purpose:**

We developed a new approach to visualize skin Langerhans cells by in vivo fluorescence imaging in nonhuman primates.

**Procedures:**

Macaques were intradermally injected with a monoclonal, fluorescently labeled antibody against HLA-DR molecule and were imaged for up to 5 days by fibered confocal microscopy (FCFM).

**Results:**

The network of skin Langerhans cells was visualized by in vivo fibered confocal fluorescence microscopy. Quantification of Langerhans cells revealed no changes to cell density with time. Ex vivo experiments confirmed that injected fluorescent HLA-DR antibody specifically targeted Langerhans cells in the epidermis.

**Conclusions:**

This study demonstrates the feasibility of single-cell, in vivo imaging as a noninvasive technique to track Langerhans cells in nontransgenic animals.

## 1. Introduction

Langerhans cells (LCs) are the subset of myeloid dendritic cells that populate the epidermal layer of the skin. As professional antigen presenting cells, LCs play an important role in first line of defense by continuously scanning the tissue for foreign antigens. After activation, LCs leave the epidermis and shuttle cutaneous antigens to the draining lymph node where adaptive immune responses are initiated [1]. The quantity and the quality of external signals determine the functional role of LCs during immune responses and direct these cells towards immunogenic or tolerogenic patterns [2]. Thus, LCs are involved in contact hypersensitivity [3], vaccination mechanisms [4, 5], graft-versus-host diseases [6, 7], tumor immunotherapy responses [8], autoimmune diseases [9], and the suppression of cutaneous infections [10]. Therefore, LCs play a pivotal role in immune regulation, and their involvement in each immune mechanism should be thoroughly investigated.

Classical ex vivo approaches have been used to characterize phenotypic markers and the functional properties of LCs [11]. However, there is an increasing necessity to explore the behavior of LCs in their natural environment in real time. With the development of transgenic mouse models expressing fluorescent proteins under the control of promoters of genes expressed specifically in the dendritic cells, such as CD11c [12] and Langerin [13], it has become possible to visualize the dynamic behavior of LCs in vivo. In vivo fluorescence imaging studies involving two-photon microscopy, mostly performed in mice, have revealed changes to the motility of LCs, antigen uptake, and interactions with other immune cells that occur in inflammatory conditions [5, 14, 15]. However, these noninvasive approaches for visualizing cells cannot be directly transferred to large animals, which may be more relevant for modeling responses to human vaccines and treatments.

Fibered confocal fluorescence microscopy (FCFM) is a suitable imaging tool for cell tracking studies because it records fluorescent signal at single-cell resolution and it is not limited to small animals. FCFM has been widely used in mice, for drug and nanoparticle distribution studies [16–18], tumor angiogenesis imaging [19], DNA fragmentation visualization [20], and the diagnosis of cancer and infections [21–23]. FCFM has also been used to examine the microstructural organization of various tissues in humans, including the skin [24, 25], cervix [26], and gastrointestinal tract [27, 28].

Here, we used FCFM as a noninvasive method to visualize skin antigen presenting cells in nonhuman primates (NHP). We demonstrate a new approach to visualize and to quantify the density of the LC network in vivo over the course of several days. This method can be applied to study LC behavior in inflammatory conditions in NHPs, which may be highly relevant to assess the efficacy of vaccines and therapeutic approaches targeting human skin.

## 2. Materials and Methods

### 2.1. Animals

All in vivo imaging studies were performed on adult cynomolgus macaques (*Macaca fascicularis*), imported from Mauritius breeding farm and weighing 3–6 kg, were housed in CEA facilities (accreditation: C 92-032-02), and were handled in accordance with European directive on protection of animals used for scientific purposes guidelines for NHP care (EU Directive N 63/2010). This study was approved by the regional committee for animal care and use (Comité d'Ethique en Expérimentation Animale number 44, AP number 11_008). Animals were handled under sedation with an intramuscular injection of 10 mg/kg ketamine hydrochloride (Merial, France).

### 2.2. Preparation of Fluorescent-Labeled Antibodies

Purified monoclonal antibodies against HLA-DR molecules (clone L243) and its isotype control (IgG2a) were purchased from Ozyme (St Quentin en Yvelines, France) and covalently conjugated to fluorochromes with FluoProbe 490 or 647 labeling kits (Interchim, Montluçon, France), via the -NHS ester. The Nanodrop microvolume spectrometer system (Thermo Scientific, Waltham, USA) was used to measure protein and fluorochrome concentrations of the purified conjugated antibodies. The Zenon® kit (Molecular Probes, Invitrogen) was used to label pure monoclonal CD1a antibody (clone 010) from Dako (Les Ulis, France) with Alexa Fluor 488, which was used immediately after conjugation.

### 2.3. In Vivo Fibered Confocal Fluorescence Microscopy

**Figure 1:**
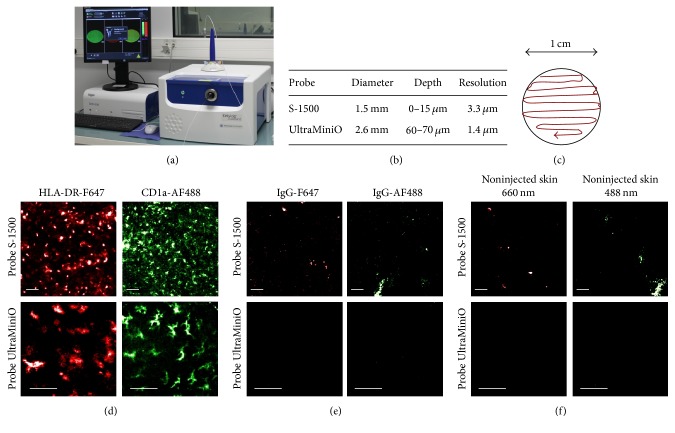
FCFM imaging of the skin. FCFM system (a). Characteristics of the probes (b). Continuous scanning was performed manually on imaged area that covers a diameter of 1 cm (c). Representative in vivo images of the skin performed 30 min after injection of anti-HLA-DR-F647 or anti-CD1a-AF488 antibodies (d); corresponding isotype controls (e). Untreated skin (f) (*n* = 3). Scale bars: 50 *μ*m.

Skin hair was removed with cold wax and skin surface was carefully cleaned to eliminate all remaining debris. Fluorescent antibodies (HLA-DR and/or CD1a) were prepared in 80 *μ*L of sterile phosphate buffered saline (PBS) solution and administered by single intradermal (i.d.) injection into macaques under anesthesia. Images were recorded from 30 minutes after injection. A dose of 5 *μ*g fluorochrome-labeled antibody was sufficient to obtain a stable signal for at least 48 h of imaging. For the longitudinal study of LC tracking, fluorescently labeled HLA-DR was injected twice: one injection 4 h before the beginning of in vivo imaging and the second injection 49 h after the first one. In vivo longitudinal imaging was performed daily for 5 days by fibered confocal fluorescence microscopy (FCFM) (Cellvizio® Dual Band, Mauna Kea Technologies, France) (Figure 1(a)). Prior to imaging, the Cellvizio® system was calibrated to adjust the fluorescence to background ratio and homogenize the detection capacity of individual fibers. Two different microprobes were used for imaging studies: the probe S-1500 and the probe UltraMiniO (Figure 1(b)). Microprobes were applied directly to the skin surface and moved continuously to record the fluorescent signal distribution all over the area of interest. Cutaneous fluorescently labeled cells were visualized in an area with a diameter of 1 cm around the site of injection (Figure 1(c)).

### 2.4. Quantification of Langerhans Cells

Twenty representative images per animal were then randomly chosen from the whole region of interest for each time point to assess the distribution of Langerhans cells in the skin. An automatic threshold algorithm was applied to filter the background signal with Image J software (National Institute of Mental Health, Bethesda, USA). The number of fluorescent objects, with a size above 4.5 *μ*m, was quantified after binary transformation of each selected frame.

### 2.5. Immunohistofluorescence of Skin Sections

Skin biopsies were performed 2 hours after the intradermal administration of fluorescently labeled antibodies, embedded in OCT and frozen in liquid nitrogen. Ten micrometer sections were fixed in 4% of PFA for 15 min and cell nucleus was stained with DAPI. Images were acquired with the Leica SP5 confocal microscope system.

### 2.6. Flow Cytometry

Skin biopsies (8 mm in diameter) were performed 2 hours after antibody injection. The tissue was mechanically dissociated (gentleMACS™, Miltenyi Biotec, Paris) and a cell suspension was obtained after 30 min of enzymatic treatment with 2 mg/mL of Collagenase D (Roche Diagnostics, France). Cells were filtered through a 70 *μ*m nylon mesh and washed with PBS. Dead cell staining was performed with LIVE/DEAD Fixable Blue Dead Cell Stain Kit (Life Technologies Invitrogen, St Aubin, France) according to the manufacturer's instructions. Cells were stained with HLA-DR-V500 antibody (clone G46-6, BD Biosciences, France) for 30 min at 4°C. Data were acquired on Fortessa (BD Biosciences, Le Pont de Claix, France) and analyzed with FlowJo software 9.4.11 (Tree Star, Ashland, OR, USA).

### 2.7. Statistical Analysis

Data are reported as means ± standard error of the mean (SEM). Pairwise comparisons were carried out in Prism 5.0 (Graph-Pad Software Inc., La Jolla, CA, USA) software with nonparametric Mann–Whitney unpaired tests.

## 3. Results

### 3.1. In Vivo Imaging of Skin Antigen Presenting Cells

We observed a homogenous network of fluorescently tagged cells after the intradermal injection of a monoclonal antibody targeting HLA-DR or CD1a molecules, the latter being a specific marker of Langerhans cells [29] (Figure 1(d)). After the injection of nonspecific antibodies, we observed a few areas of weak fluorescence with both lasers (Figure 1(e)). This was probably due to autofluorescent areas on the skin surface because the images obtained on untreated skin were similar (Figure 1(f)). We observed a regular, organized network of fluorescently labeled epidermal cells over the whole imaged area with the S-1500 probe. Higher resolution images were obtained with the probe UltraMiniO, but the fluorescent network of Langerhans cells was visible only in distinct regions of the skin. This probe allows imaging at a depth of 60 *μ*m, which corresponds to the epidermal junction, where Langerhans cells are situated mostly in the hair follicle. Thus, the S-1500 probe was retained to evaluate the density of Langerhans cells by in vivo imaging.

### 3.2. Use of HLA-DR Antibody for the Visualization of Langerhans Cells In Vivo

**Figure 2:**
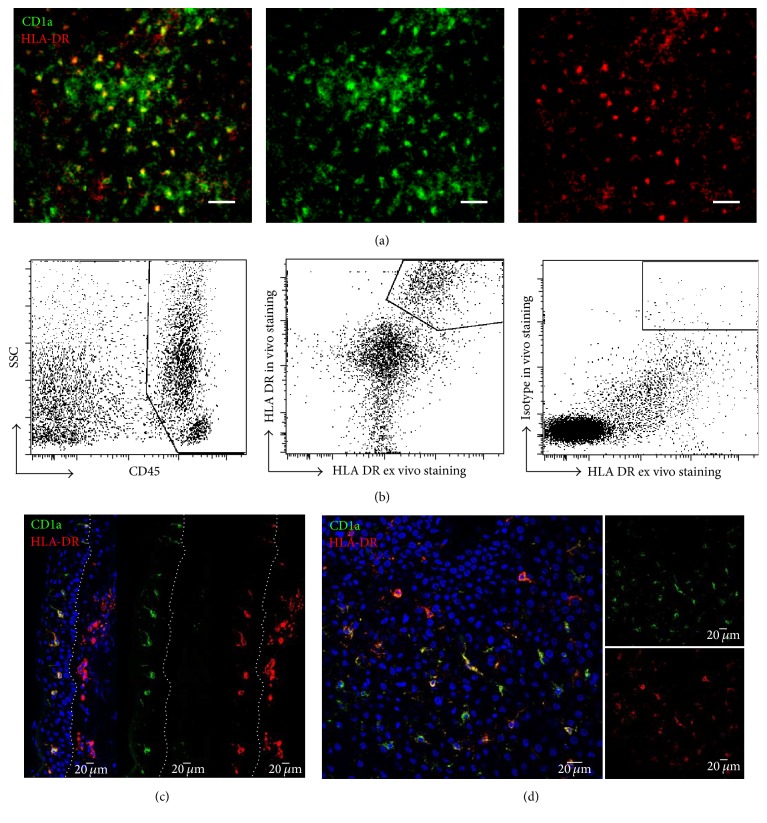
HLA-DR antibody for LC in vivo tracking. In vivo (a) representative images of Langerhans cells 2 h after in vivo injection of monoclonal antibodies HLA-DR-F647 and CD1a-AF488 (*n* = 3). Scale bars: 50 *μ*m. (b) Flow cytometry analysis of fluorescently labeled immune cells isolated from skin biopsy 2 h after i.d. injection of monoclonal fluorescently labeled HLA-DR antibody (clone L243) or its isotype control. Skin cell suspensions were stained ex vivo with CD45 and HLA-DR (clone G46-6) antibodies. Transversal (c) section of skin biopsies and longitudinal (d) section of epidermis frozen 2 h after injection of fluorescent antibodies. Dotted lines split epidermal (left) and dermal (right) layers.

We have previously shown ex vivo that CD1a+ cells are the only HLA-DR+ population in the epidermis of macaque [30]. Here CD1a antibody was coadministrated intradermally with HLA-DR antibody to confirm the specificity of LC labeling in vivo. In vivo FCFM (Figure 2(a)) showed that HLA-DR-labeled epidermal cells were also labeled with CD1a antibody. Flow cytometry analyses of the CD45^+^ leukocyte population isolated from both the dermis and epidermis further confirmed the cell binding specificity of in vivo injected HLA-DR antibody, whereas nonspecific antibody did not appear to bind to skin cells when injected in vivo (Figure 2(b)). Microscopic analysis of frozen skin samples demonstrated epidermal and dermal distribution of HLA-DR antibody on transversal skin sections (Figure 2(c)). In the epidermis, all HLA-DR labeled cells were equally labeled with CD1a antibody (Figure 2(d)).

### 3.3. Longitudinal In Vivo Imaging of Langerhans Cells in Macaque Skin

**Figure 3:**
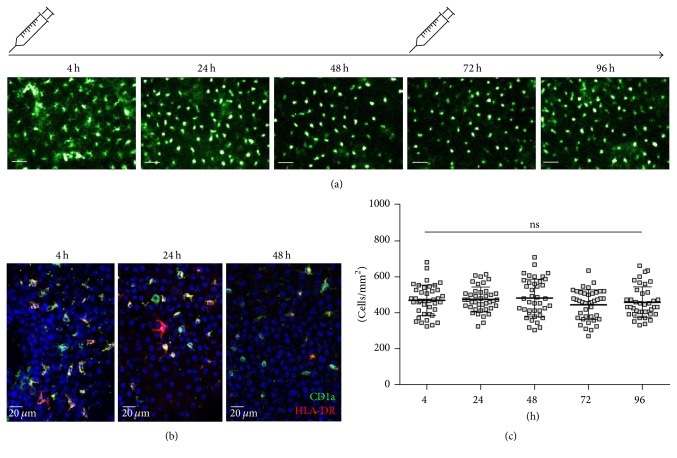
In vivo imaging and quantification of LCs. (a) Images of HLA-DR-labeled Langerhans cells in macaque epidermis were obtained by in vivo fibered confocal microscopy over 5 days. Scale bars: 50 *μ*m. (b) Immunohistofluorescence on longitudinal sections of epidermis from skin biopsies obtained 4 h, 24 h, or 48 h after injection of HLA-DR and CD1a antibodies. (c) Quantification of the cell number from 20 different frames/animal that were randomly chosen from videos covering an imaging zone of 1 cm in diameter (*n* = 2). Mean ± SEM, Friedman's test. ns: nonsignificant. Representative images are shown for one from 2 analyzed animals.

We imaged the Langerhans cell network in the epidermis at the antibody injection site every day for 5 days (Figure 3(a)). The fluorescent signal remained stable for 48 h (Figure 3(b)); thus two injections of fluorescent antibody were sufficient to cover the imaging duration. The number of Langerhans cells in the epidermis remained constant over time of imaging (Figure 3(c)).

## 4. Discussion

In vivo fluorescence imaging tools have been developed and adapted to various immunological contexts in small animal models. The behavior of LCs in vivo has been largely studied by intravital microscopy. Most studies have used genetically modified mice in which distinct cell populations express fluorescent proteins [12, 31, 32]. Moreover, topical application of fluorescent solutions, known as skin painting, results in the unspecific uptake of fluorescent dye by phagocytic skin cells, thus allowing studies on LC migration to the draining lymph node [33, 34]. In vivo imaging of cutaneous dendritic cells has also been performed after the intradermal injection of vital dye (CFSE) [35] or after the subcutaneous injection of Quantum Dots [36]. Moreover, we [37] and others have tracked bone marrow-derived dendritic cells, that present dendritic cell phenotype and functions; however it is not expected that these cells behave as Langerhans cells even if they are injected into the skin. Here, we described a new approach for the noninvasive imaging of Langerhans cells in macaques. We show that a small quantity of locally injected fluorescent antibody provides a specific and stable signal for longitudinal FCFM studies. The use of various microprobes on the tissue surface allows the noninvasive visualization of cellular events at various depths with this microscopy approach. However, only the S-1500 probe revealed the organization of the LC network in the whole imaged region. The thickness of the macaque epidermis varies between 15 *μ*m and 33 *μ*m, depending on the anatomical site [38]. Given the technical parameters of the UltraMiniO probe (60 *μ*m working distance), the signal detected with this probe should be situated in the upper layer of the dermis. This was concordant with our observations of the zonal distribution of LCs probably situated in hair follicles. Variations in the thickness of the epidermis between species should also be taken into account when using FCFM. For example, in the mouse, the epidermis is between 7 and 15 *μ*m thick [38] and the S-1500 probe is very appropriate to visualize LCs, whereas, in human skin, the UltraMiniO probe is better adapted to image the epidermal layer, which usually exceeds 50 *μ*m in thickness [39].

Fibered confocal microscopy allows the longitudinal tracking of immune cells and their quantification may indicate pertinent time points for analysis involving invasive procedures and thus help to limit the number of biopsies per animal. Other strategies involving fluorescence imaging such as two-photon microscopy offer the possibility of studying cell mobility and behavior at greater depths than FCFM. However, these systems are mainly dedicated to small animal models, whereas FCFM is very adaptable for use in large animals.

HLA-DR is highly expressed on the surface of antigen presenting cells, which makes it a good target for imaging experiments. We have previously shown that, in the epidermal layer of macaque skin, the only cell population that expresses HLA-DR marker is the population of LCs [30]. However, HLA-DR expression is not limited to LC; therefore, the use of a more specific LC markers such as CD1a [37] enables LCs to be differentiated from other antigen presenting cells. Here, we demonstrated that the HLA-DR antibody binds to CD1a positive cells in the epidermis in the steady state in vivo and that its isotype control is not taken up by nonspecific Fc-mediated uptake. Moreover, flow cytometry analysis confirmed that intradermally injected HLA-DR antibody specifically binds HLA-DR-expressing skin cells.

However, the incubation of epidermal cells with the HLA-DR antibody in vitro leads to the internalization of the antibody through common receptor-mediated endocytosis [40], which may potentially induce cell activation. The activation of LCs often results in their migration from the epidermal layer to the draining lymph node [4]. In this study, LC density observed by in vivo imaging remained unchanged over time, suggesting that in vivo antibody injection, in this experimental setup, did not interfere with LC migration. This is consistent with previous reports showing that the intradermal injection of monoclonal antibodies targeting LCs, in the absence of costimulation signals such as the Toll-Like Receptor Ligand agonist, does not induce LC activation [41, 42].

Here, we used less than 2 *μ*g of antibody per kg of body weight/injection and its intradermal administration was not associated with signs of local inflammation. However, many repeated injections should be avoided because this can stimulate memory immune responses leading to the degradation of the imaging tracer and a decrease in fluorescent signal. Fab antibody fragments may be used for the targeting and labeling of immune cells in vivo to limit the risk of immune activation. Although immune cell imaging has been successfully performed with Fab fragments in mice [43, 44], their fluorochrome binding is often less bright than that of classical antibodies because of their smaller size [45].

## 5. Conclusion

In this report, we describe the use of fibered confocal fluorescence microscopy as a new method to visualize Langerhans cells in the macaque epidermis in vivo. We show that intradermally injected fluorescent antibodies against HLA-DR molecules specifically bind to LCs in the epidermis and provide a bright and stable fluorescent signal for longitudinal studies. We also quantified cell density in the area of interest. Thus, this approach may be used to study noninvasively the changes in LC density in various immunological environments in large animal models.

## Abbreviations

LC: Langerhans cells
i.d.: Intradermal
FCFM: Fibered confocal fluorescence microscopy
NHP: Nonhuman primates
i.m.: Intramuscular.

## References

[1] Romani N., Clausen B. E., Stoitzner P. (2010). Langerhans cells and more: langerin-expressing dendritic cell subsets in the skin. *Immunological Reviews*.

[2] Seneschal J., Clark R. A., Gehad A., Baecher-Allan C. M., Kupper T. S. (2012). Human epidermal Langerhans cells maintain immune homeostasis in skin by activating skin resident regulatory T cells. *Immunity*.

[3] Bobr A., Olvera-Gomez I., Igyarto B. Z., Haley K. M., Hogquist K. A., Kaplan D. H. (2010). Acute ablation of Langerhans cells enhances skin immune responses. *Journal of Immunology*.

[4] Liard C., Munier S., Joulin-Giet A. (2012). Intradermal immunization triggers epidermal langerhans cell mobilization required for CD8 T-cell immune responses. *Journal of Investigative Dermatology*.

[5] Rattanapak T., Birchall J. C., Young K. (2014). Dynamic visualization of dendritic cell-antigen interactions in the skin following transcutaneous immunization. *PLoS ONE*.

[6] Kreutz M., Karrer S., Hoffmann P. (2012). Whole-body UVB irradiation during allogeneic hematopoietic cell transplantation is safe and decreases acute graft-versus-host disease. *Journal of Investigative Dermatology*.

[7] Merad M., Hoffmann P., Ranheim E. (2004). Depletion of host Langerhans cells before transplantation of donor alloreactive T cells prevents skin graft-versus-host disease. *Nature Medicine*.

[8] Stoitzner P., Green L. K., Jung J. Y. Tumor immunotherapy by epicutaneous immunization requires langerhans cells. *The Journal of Immunology*.

[9] Shaw F. L., Cumberbatch M., Kleyn C. E. (2010). Langerhans cell mobilization distinguishes between early-onset and late-onset psoriasis. *Journal of Investigative Dermatology*.

[10] Kautz-Neu K., Noordegraaf M., Dinges S. (2011). Langerhans cells are negative regulators of the anti-*Leishmania* response. *The Journal of Experimental Medicine*.

[11] Helft J., Ginhoux F., Bogunovic M., Merad M. (2010). Origin and functional heterogeneity of non-lymphoid tissue dendritic cells in mice. *Immunological Reviews*.

[12] Lindquist R. L., Shakhar G., Dudziak D. (2004). Visualizing dendritic cell networks in vivo. *Nature Immunology*.

[13] Kissenpfennig A., Malissen B. (2006). Langerhans cells - Revisiting the paradigm using genetically engineered mice. *Trends in Immunology*.

[14] Sen D., Forrest L., Kepler T. B., Parker I., Cahalan M. D. (2010). Selective and site-specific mobilization of dermal dendritic cells and Langerhans cells by Th1- and Th2-polarizing adjuvants. *Proceedings of the National Academy of Sciences of the United States of America*.

[15] Celli S., Albert M. L., Bousso P. (2011). Visualizing the innate and adaptive immune responses underlying allograft rejection by two-photon microscopy. *Nature Medicine*.

[16] Derieppe M., Yudina A., Lepetit-Coiffé M., De Senneville B. D., Bos C., Moonen C. (2013). Real-time assessment of ultrasound-mediated drug delivery using fibered confocal fluorescence microscopy. *Molecular Imaging and Biology*.

[17] Mahe B., Vogt A., Liard C. (2009). Nanoparticle-based targeting of vaccine compounds to skin antigen-presenting cells by hair follicles and their transport in mice. *Journal of Investigative Dermatology*.

[18] Bai J., Wang J. T., Mei K. (2016). Real-time monitoring of magnetic drug targeting using fibered confocal fluorescence microscopy. *Journal of Controlled Release*.

[19] Fitoussi V., Faye N., Chamming's F., Clement O., Cuenod C.-A., Fournier L. S. (2013). In vivo imaging of tumor angiogenesis using fluorescence confocal videomicroscopy. *Journal of Visualized Experiments*.

[20] Al-Gubory K. H. (2005). Fibered confocal fluorescence microscopy for imaging apoptotic DNA fragmentation at the single-cell level in vivo. *Experimental Cell Research*.

[21] Eser S., Messer M., Eser P. (2011). In vivo diagnosis of murine pancreatic intraepithelial neoplasia and early-stage pancreatic cancer by molecular imaging. *Proceedings of the National Academy of Sciences of the United States of America*.

[22] Morisse H., Heyman L., Salaün M. (2013). In vivo molecular microimaging of pulmonary aspergillosis. *Medical Mycology*.

[23] Elahi S. F., Liu Z., Luker K. E., Kwon R. S., Luker G. D., Wang T. D. (2011). Longitudinal molecular imaging with single cell resolution of disseminated ovarian cancer in mice with a LED-based confocal microendoscope. *Molecular Imaging and Biology*.

[24] Swindle L. D., Thomas S. G., Freeman M., Delaney P. M. (2003). View of Normal Human Skin In Vivo as Observed Using Fluorescent Fiber-Optic Confocal Microscopic Imaging. *Journal of Investigative Dermatology*.

[25] Suihko C., Serup J. (2012). Fluorescent fibre-optic confocal imaging of lesional and non-lesional psoriatic skin compared with normal skin in vivo. *Skin Research and Technology*.

[26] Tan J., Quinn M. A., Pyman J. M., Delaney P. M., McLaren W. J. (2009). Detection of cervical intraepithelial neoplasia in vivo using confocal endomicroscopy. *BJOG: An International Journal of Obstetrics and Gynaecology*.

[27] Polglase A. L., McLaren W. J., Skinner S. A., Kiesslich R., Neurath M. F., Delaney P. M. (2005). A fluorescence confocal endomicroscope for in vivo microscopy of the upper- and the lower-GI tract. *Gastrointestinal Endoscopy*.

[28] Atreya R., Neumann H., Neufert C. (2014). In vivo imaging using fluorescent antibodies to tumor necrosis factor predicts therapeutic response in Crohn's disease. *Nature Medicine*.

[29] Epaulard O., Adam L., Poux C. (2014). Macrophage-and neutrophil-derived TNF-*α* instructs skin Langerhans cells to prime antiviral immune responses. *Journal of Immunology*.

[30] Adam L., Rosenbaum P., Cosma A., Le Grand R., Martinon F. (2015). Identification of skin immune cells in non-human primates. *Journal of Immunological Methods*.

[31] Vishwanath M., Nishibu A., Saeland S. (2006). Development of intravital intermittent confocal imaging system for studying Langerhans cell turnover. *Journal of Investigative Dermatology*.

[32] Kissenpfennig A., Henri S., Dubois B. (2005). Dynamics and function of Langerhans cells in vivo: dermal dendritic cells colonize lymph node areas distinct from slower migrating Langerhans cells. *Immunity 22*.

[33] Eidsmo L., Allan R., Caminschi I., Van Rooijen N., Heath W. R., Carbone F. R. (2009). Differential migration of epidermal and dermal dendritic cells during skin infection. *Journal of Immunology*.

[34] Furmanov K., Elnekave M., Lehmann D., Clausen B. E., Kotton D. N., Hovav A.-H. (2010). The role of skin-derived dendritic cells in CD8+ T cell priming following immunization with lentivectors. *Journal of Immunology*.

[35] Miller M. J., Hejazi A. S., Wei S. H., Cahalan M. D., Parker I. (2004). T cell repertoire scanning is promoted by dynamic dendritic cell behavior and random T cell motility in the lymph node. *Proceedings of the National Academy of Sciences of the United States of America*.

[36] Sen D., Deerinck T. J., Ellisman M. H., Parker I., Cahalan M. D. (2008). Quantum dots for tracking dendritic cells and priming an immune response in vibtro and in vivo. *PLoS ONE*.

[37] Romain G., van Gulck E., Epaulard O. (2012). CD34-derived dendritic cells transfected ex vivo with HIV-Gag mRNA induce polyfunctional T-cell responses in nonhuman primates. *European Journal of Immunology*.

[38] Monteiro-Riviere N. A., Bristol D. G., Manning T. O., Rogers R. A., Riviere J. E. (1990). Interspecies and interregional analysis of the comparative histologic thickness and laser Doppler blood flow measurements at five cutaneous sites in nine species. *Journal of Investigative Dermatology*.

[39] Robertson K., Rees J. L. (2010). Variation in epidermal morphology in human skin at different body sites as measured by reflectance confocal microscopy. *Acta Dermato-Venereologica*.

[40] Hanau D., Fabre M., Schmitt D. A. (1987). Human epidermal Langerhans cells cointernalize by receptor-mediated endocytosis 'nonclassical' major histocompatibility complex class I molecules (T6 antigens) and class II molecules (HLA-DR antigens). *Proceedings of the National Academy of Sciences of the United States of America*.

[41] Flacher V., Tripp C. H., Stoitzner P. (2010). Epidermal langerhans cells rapidly capture and present antigens from C-type lectin-targeting antibodies deposited in the dermis. *Journal of Investigative Dermatology*.

[42] Salabert N., Todorova B., Martinon F. (2016). Intradermal injection of an anti-Langerin-HIVGag fusion vaccine targets epidermal Langerhans cells in nonhuman primates and can be tracked in vivo. *European Journal of Immunology*.

[43] Moreau H. D., Lemaître F., Terriac E. (2012). Dynamic in situ cytometry uncovers T cell receptor signaling during immunological synapses and kinapses in vivo. *Immunity*.

[44] Garcia Z., Lemaître F., van Rooijen N. (2012). Subcapsular sinus macrophages promote NK cell accumulation and activation in response to lymph-borne viral particles. *Blood*.

[45] Progatzky F., Dallman M. J., Celso C. L. (2013). From seeing to believing: Labelling strategies for in vivo cell-tracking experiments. *Interface Focus*.

